# Supplementary material for: Predicting visual function by interpreting a neuronal wiring diagram
Source: Nature. 2024 Oct 2;634(8032):113–23. doi: 10.1038/s41586-024-07953-5 (PMC11446822; doi:10.1038/s41586-024-07953-5)
Supplement: Supplementary file 6 — CRF and ERF predictions for individual TmY4 and TmY9 cells. Analogous to Supplementary Data 3, but for TmY target types. Shown are the top four monosynaptic pathways, the strongest pathway passing through each of the top ten intermediary types (ranking from Extended Data Fig. 7), and the trisynaptic pathway Tm1–TmY–Dm3–TmY (see the section entitled Prediction of spatial normalization). [file 41586_2024_7953_MOESM6_ESM.zip › DataS4/TmY9q/720575940614402466.pdf]

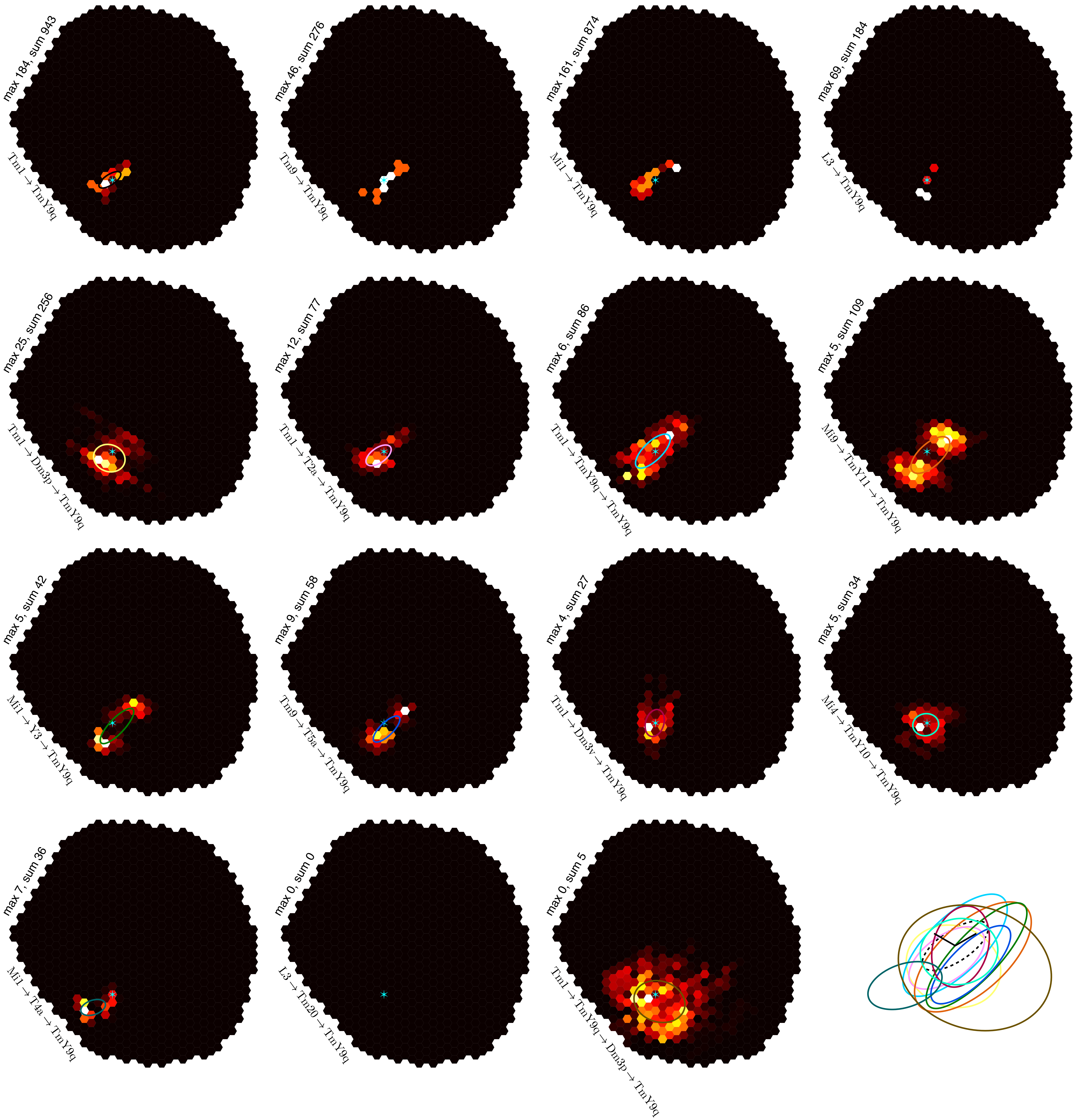
max 184, sum 943
Tm1 → TmY9q
max 46, sum 276
Tm9 → TmY9q
max 161, sum 874
Mi1 → TmY9q
max 69, sum 184
L3 → TmY9q
max 25, sum 256
Tm1 → Dm3p → TmY9q
max 12, sum 77
Tm1 → T2a → TmY9q
max 6, sum 86
Tm1 → TmY9q → TmY9q
max 5, sum 109
Mi9 → TmY11 → TmY9q
max 5, sum 42
Mi1 → Y3 → TmY9q
max 9, sum 58
Tm9 → T5a → TmY9q
max 4, sum 27
Tm1 → Dm3v → TmY9q
max 5, sum 34
Mi4 → TmY10 → TmY9q
max 7, sum 36
Mi1 → T4a → TmY9q
max 0, sum 0
L3 → Tm20 → TmY9q
max 0, sum 5
Tm1 → TmY9q → Dm3p → TmY9q
